# How COVID-19 Changed the Information Needs of Italian Citizens

**DOI:** 10.3390/ijerph17196988

**Published:** 2020-09-24

**Authors:** Rino Falcone, Alessandro Sapienza

**Affiliations:** Institute of Cognitive Sciences and Technologies, ISTC-CNR, 00185 Rome, Italy; rino.falcone@istc.cnr.it

**Keywords:** COVID-19, SARS-CoV-2, coronavirus, fake news, misleading information, trust, misinformation, information-seeking behavior, social simulation

## Abstract

Italy was the first European country to be affected by COVID-19, facing an unprecedented situation. The reaction required drastic solutions and highly restrictive measures, which severely tested the trust of the Italian people. Nevertheless, the effectiveness of the introduced measures was not only linked to political decisions, but also to the choice of the Italian people to trust and rely on institutions, accepting such necessary measures. In this context, the role of information sources was fundamental, since they strongly influence public opinion. The central focus of this research was to assess the information seeking behavior (ISB) of the Italian citizens, to understand how they related to information and how their specific use of information influenced public opinion. By making use of a survey addressed to 4260 Italian citizens, we identified extraordinarily virtuous behavior in the population: people strongly modified their ISB in order to address the most reliable sources. In particular, we found a very high reliance on scientists, which is particularly striking, if compared to the past. Moreover, starting from the survey results, we used social simulation to estimate the evolution of public opinion. Comparing the ISB during and before COVID-19, we discovered that the shift in the ISB, during the pandemic, may have actually positively influenced public opinion, facilitating the acceptance of the costly restrictions introduced.

## 1. Introduction

**Figure 1:**
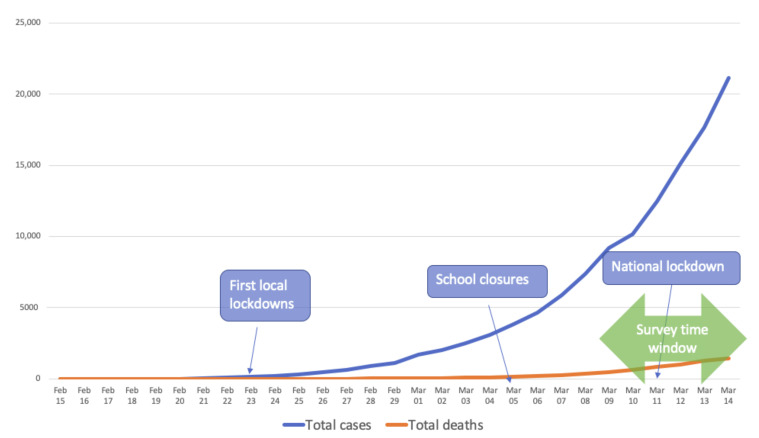
Evolution of COVID-19 in Italy.

The COVID-19 outbreak forced the Italian State to deal with a novel, ambiguous, and unexpected risk. The first cases were reported in late February and in a few weeks the pandemic spread rapidly throughout the rest of the country. Politicians, local governments, and citizens had to face an unprecedented situation. Likewise, the whole scientific community promptly took action to provide all possible support and to help find solutions to save lives and reduce strain on the health care system. An exceptional situation calls for exceptional measures. The reaction to COVID-19 in Italy required drastic solutions and highly restrictive measures [1]. Consider that, on March 11, Italy was the first European country entering a nationwide lockdown (see Figure 1).

In this context of uncertainty and constant change, a strong need for information emerged [2], in order to understand what was going on: how the pandemic was evolving; to assess the actual risk to which all of us were subjected; to know community-level policies or personal health strategies. Quarantine, social distancing, mass swab tests, school closures, and the use of personal protective equipment are just some of the highly restrictive measures adopted to deal with the pandemic. People were required to undertake costly behavioral changes, not only in economic terms, but even restricting their personal freedom. All this was accompanied by a strong sense of uncertainty about the future. As Gualano and colleagues state [3], the Italian general population reported a high prevalence of mental health issues during the lockdown (depression, anxiety, poor sleep), whose impact is expected to persist beyond this critical situation.

The trust of the Italian people has been severely tested, since, while some of these restrictions are still ongoing, uncertainties regarding COVID-19 make it difficult to evaluate their effectiveness. Nevertheless, establishing these costly and drastic measures would not have been enough to face the pandemic. Such measures would have been vain if a significant percentage of the population had considered them inadequate or inappropriate, not respecting them. The institutions needed the trust of the Italian population, and indeed, in a time of crisis, the Italian population decided to trust its institutions and to rely on them to face COVID-19.

This scenario was anything but trivial, considering the historical distrust in the Italian government and institutions in general. A survey on a representative sample of Italian citizens (N = 1028, 16–17 March 2020), conducted by the independent research center Demos and Pi (http://www.demos.it/a01705.php, accessed at 07/08/2020) in the same period that we consider in this study, reported that 71% of citizens trust both the Italian government and the current prime minister, compared to the 44% of the previous month, and 94% approval of the adopted measures. The recent data of the Eurispes Report—Italy 2020 (https://eurispes.eu/news/eurispes-risultati-del-rapporto-italia-2020/, accessed at 07/08/2020), presented in February 2020, indicated trust in institutions at 14.6% (6.2 points lower than in 2019). According to the analysis conducted by Statista (https://www.statista.com/statistics/977223/support-for-prime-minister-conte-in-italy/, accessed at 07/08/2020), trust in the Italian prime minister in February was around 39%. The same report shows a sudden rise of trust up to 54% immediately after the emergency arrival. For sure, it is unthinkable that the authorities have suddenly become more reliable, but rather the citizens were forced by the circumstances. They had no other choice but to rely on their institutions, to trust the only entity able to face the problem, accepting all the necessary rules and restrictions [4]. The high level of trust in the Italian institutions about COVID-19 may have considerably contributed to the successful management of the pandemic.

Nevertheless, such a necessary choice may have resulted in a series of adjustments, in order to compensate and justify this "trust gap" [4]. As it is well known in the literature [5], in such cases, feedback and control mechanisms come into play. When there is not enough trust, making use of some type of control on the trustee allows one to lower the level of trust needed for reliance. From this consideration followed the great needs to get information, to monitor the institutions, to know that trust was well placed, and that the sacrifices the citizens were forced to make were fundamental. As Siegrist and Zingg report [6] in their systematic review on the importance of trust when preparing for and during a pandemic, trust is fundamental to positively influencing people’s willingness to adopt recommended behavior.

Therefore, the promptness of the applied measures (closures, limitations, personal protective equipment, interpersonal distance) would not have been enough to limit the impact of this pandemic, but it was also necessary to have a proper information dissemination process. In these cases, trust in institutions is therefore strongly linked to their communication skills, and to the fact that they are able to demonstrate the effectiveness of the proposed strategies. Even the World Health Organization, in a 2011 report [7], has identified communication as one of the biggest challenges, among the essential instruments required to tackle a pandemic. Our own opinions and beliefs are strictly tied to information we receive, especially in a moment of total uncertainty [8,9,10]; consider the lack of prior knowledge about this virus. This becomes particularly critical when, on the one hand there is an enormous risk, and on the other there is even the limitation of one’s personal freedoms. Our trust is strongly related to information we receive and a lack of trust could lead to the non-acceptance of the rules imposed, the actual result of which depends not on the acceptance of the individual, but on the adhesion of a substantial part of the population. In such a scenario, it is clear how fundamental it is to inform citizens properly, by identifying the right communication methods and limiting the spread of misinformation. As an example, manifold fake news [11] about COVID-19 were spread, probably aiming to affect public health communication and diminish preventive measures. The main, but not exclusive source of this fake news was social media. In order to answer this situation, the Italian Ministry of Health was forced to change its communication strategies, playing a strategic role in using its official Facebook page to mitigate the spread of misinformation and to offer updates to the online public [12]. Overall, the authorities’ response to fake news was effective.

Another problem that arose, with respect to information, concerned the not always complete consistency of the indications provided by official sources (the various experts: virologists, infectious disease specialists, pandemic phenomena experts, etc.) as the pandemic proceeded. This phenomenon is well known to science, as many problems typically require a certain amount of time to be studied and to produce stable knowledge. Nevertheless, the population is not accustomed to such processes. Due to constant media pressure, motivated by the necessity to investigate the different perspectives of the pandemic, there has been an overexposure of experts in public debates. In several cases, citizens were faced with rapidly changing hypotheses, which at times were also contradictory to each other. (Has the virus lost spreading and infectious capabilities or not? Can the asymptomatic subjects transmit the virus or not?)

### Research Questions

The study presented in this paper contributes to this fast-growing body of knowledge on the interplay between trust in institutions and the COVID-19 pandemic, by discussing the results of a large scale survey (N = 4260) conducted on Italian citizens between 9 March and 14 March 2020, and already analyzed to evaluate the aspects of trust in institutions [4]. The survey, theoretically inspired by the socio-cognitive model of trust developed by Castelfranchi and Falcone [13], aimed to take a closer look at the cognitive and social factors responsible for trust towards public institutions in the face of pandemic threats. Starting from the results of the previous study (trust boom, trust gap, and the consequent need to exercise some form of control), within this paper we aim to assess how the citizens’ information-seeking behavior influenced the perception of government response strategies during the pandemic. Thus, we investigate how the Italian population related to information and information sources during the early stages of the pandemic.

Specifically, we intend to test the hypothesis that the Italian population has behaved in a virtuous way, precisely because of the use they made of information. We believe that their virtuous behavior may have affected public opinion, by making it move towards compliance with the restrictive rules, which in turn may have reduced the impact of the pandemic.

## 2. Related Work

Information is a primary good for human beings, as it allows to reduce uncertainty and to make the world more predictable [14]. Our own opinions and beliefs are strictly tied to information we receive, especially if we are forced to face phenomena that are currently completely unknown [8,9,10]. Quality information represents the basis of good decision-making, which becomes pivotal when it comes to healthcare. Information seeking behavior (ISB) refers to those activities a person engages in when identifying his or her own need for information, searching for such information in any way, and using or transferring that information [15]. Health information seeking relates to the ways in which individuals obtain information, including information about their health, health promotion activities, risks to one’s health, and illness [16]. As McCloud and colleagues state [17], the breadth and nature of health information obtained influences the individual’s knowledge, beliefs, and attitudes toward a specific health behavior.

In the light of such considerations, the fundamental role of ISB is clear. Much attention in the literature has focused on identifying who actively seeks or who does not seek health information, the frequency of use, and satisfaction with health information seeking [18,19]. The issue to date is that not all individuals seek health information equally.

Several studies have shed the light on the importance of demographic features for understanding information seeking, such as socioeconomic and ethnic diversity [20,21]. Specifically, for what it concerns COVID-19, age and gender effects could be particularly interesting, given that they also represent the two main factors that determine the mortality for COVID-19 [22,23,24], together with the presence of pre-existing diseases.

As far as it concerns gender, many works underlined its strong effect on ISB. As Halder and colleagues [15] state, "Gender as a variable may be useful for better understanding the cognitive and social background of human information processing and may have important implications for information dissemination services and systems." The same authors, in their study, confirmed one of the well-known gender effects in the literature. They discovered that females seem to be more ardent information searchers when compared to males, and that they also have more information needs than males. Similarly, Manierre [25] found that females are more likely to look for health information, with respect to males. Such an effect also applies to online sources [26].

Much less known, however, are the effects of age, with particular reference to the elderly. Although there are some studies in the literature, most of them focus on their specific use of the Internet [27,28]. Yet, having a clear picture of older adults’ health information-seeking behavior has an evident and substantial practical value. Indeed, given that this social category is generally the most subject to health-related risks, understanding how the elderly relate to information would be of great help to minimizing the diffusion of poor or potentially threatening health information or improving the diffusion of useful health information [29].

In summary, given the great importance of the ISB in decision-making processes, it is clear that studying it is fundamental to understanding how individual citizens (and the whole community) responded to the COVID-19 emergency. These considerations would be particularly useful both for public institutions and for the healthcare system, allowing a better understanding of what happened and how public opinion could be better orientated in the future.

## 3. Materials and Methods

We report below the full details of the entire study. However, in this specific work we focus our attention on a particular subsection of the survey, taking a closer look at the information seeking behavior of the Italian citizens.

### 3.1. Sample

The study was conducted using a snowball sampling method to determine the respondents: it concerned a large sample (N = 4260, 57% women, mean age = 46 years), relatively well-balanced in terms of geographical provenance (33% Northern Italy, 39% Central Italy, 28% Southern Italy and main islands), with a significant portion of respondents (30%) residing in the regions most affected by COVID-19 at that time (Lombardy, Veneto, Emilia-Romagna, Marche, Piedmont). It should be noted that the mean educational level of participants was very high: almost three quarters of respondents had a degree (38%) or post-graduate specialization (34%). The main characteristics of the sample are shown in Table 1.

### 3.2. Survey Structure

Data were collected with a 57-item questionnaire, using a 5-point Likert scale for most items. An English translation of the whole questionnaire is available at [4].

The questionnaire was aimed to investigate the participant’s overall trust towards public authorities and their motivations, along with the factors that determine the participant’s trust. The questionnaire was based on the socio-cognitive model of trust developed by Castelfranchi and Falcone [13] and explored participants’ opinions on five main dimensions, in relation to the current COVID-19 crisis in Italy:

1. Evaluation of the competence of public institutions;
2. Evaluation of the intentionally of public institutions;
3. Purposes and effectiveness of the public institutions’ intervention;
4. Trust and information sources: the most used sources of information and their perceived trustworthiness;
5. Expectations about the future scenarios that will arise, once the COVID-19 crisis is over.

As already stated, in this work we will mainly focus on the fourth point. The questionnaire was administered online using the Google Forms platform. The questionnaire fully complied with ethical guidelines for human subject research and participation was conditional on the preliminary approval of an informed consent by each subject; the compilation took an average time of 10 min. Data analysis was performed using R-studio (version 3.6.3) statistical software, developed by RStudio, PBC (Boston, MA, USA). In particular, given the asymmetric distribution of most variables, we considered Spearman correlation values for correlation analyses.

### 3.3. Simulations

In this work, the use of simulations has allowed us to estimate how the opinions of classes of individuals and of a whole community evolved, starting from the specific use the citizens made of information sources. In order to study opinion dynamics, we considered a model based on that proposed by Hegselmann–Krause [30], whose effectiveness has been proven over time. Their model has been modified here by introducing the probabilistic use of information sources.

Then, given the belief *b* as “the institutional truth” to tackle COVID-19 outbreak (i.e., “it is essential to use face masks,” or “it is necessary to maintain social distance”), the citizens, modeled in the simulation as agents, had different opinions about said belief, since the information sources they used may have reported evidence supporting *b* or opposing it. As we supposed *b* to be the institutional truth, opposing information represents, for simplicity, misinformation and/or fake news.

Let n be the number of information sources under consideration. To model the repeated process of opinion formation, we considered a discrete-time system; thus time was divided in rounds T={0,1,2,…}. For a fixed agent *i*, we denote its opinion at time *t* by $x_{i}\left(t\right)$, expressed as a real number in [0,1]. Similarly, for a fixed information source *j*, we denote information of the sources *j* at time *t* by $inf_{j}\left(t\right)$. While fixing an agent *i*, $\delta_{i}$ represents the weight *i* gives to its own opinion, and $a_{ij}$ is the weight given to information coming from the source *j*, with 1≤j≤n.

Furthermore, let $\delta_{i}\geq 0$ and $a_{ij}\geq$ 0 for all i and j, an let their sum be equal to 1, as in Equation (2). This last condition is not mandatory, but it allows us to avoid the normalization process. Considering this notation, opinion formation of agent i can be described as in Equation (1).

(1) $$x_{i}\left(t+1\right)=\delta_{i}*x_{i}\left(t\right)+a_{i1}*inf_{1}\left(t\right)+a_{i2}*inf_{2}\left(t\right)+\cdots +a_{in}*inf_{n}\left(t\right)$$

(2) $$\delta_{i}+a_{i1}+a_{i2}+\cdots +a_{in}=1$$

In addition to the Hegselmann–Krause model, we considered that *i* would not use all its source at each round, but just a subset of them. This characteristic allowed us to model a frequency-based access to the sources, which is precisely what the citizens stated to do. Therefore, we introduce the function $use_{ij}\left(t\right)$, whose Boolean result determines if *i* will make use of the source *j* at time *t*. Thus, our model will be described by Equations (3) and (4).

(3) $$x_{i}\left(t+1\right)=\delta_{i}*x_{i}\left(t\right)+a_{i1}*inf_{1}\left(t\right)*use_{i1}\left(t\right)+a_{i2}*inf_{2}\left(t\right)*use_{i2}\left(t\right)+\cdots +a_{in}*inf_{n}\left(t\right)*use_{in}\left(t\right)$$

(4) $$\delta_{i}+a_{i1}*use_{i1}\left(t\right)+a_{i2}*use_{i2}\left(t\right)+\cdots +a_{in}*use_{in}\left(t\right)=1$$

Given this framework, we are interested in understanding how the opinion of the agent *i*, characterized by an initial profile and a specific ISB, evolves over time. By doing this analysis for each category of citizens, we are able to determine how the particular ISB of a category influences the final opinion of the citizens belonging to that category.

The data we need are:

1. Initial profile of the agent *i*, given by $x_{i}\left(0\right)$ and $\delta_{i}$;
2. ISB, i.e., $use_{ij}\left(t\right)$ and $a_{ij}$ for each information source *j*;
3. Actual trustworthiness of each information source *j*, in order to generate $inf_{j}\left(t\right)$.

As far as it concerns $x_{i}\left(0\right)$ and $\delta_{i}$, we considered a situation in which these two parameters did not affect the final outcome. Notice that, in such a system, given that information sources influence citizens but the opposite never happens, after a sufficiently wide time window $x_{i}\left(t\right)$ can be considered as independent of $x_{i}\left(0\right)$. Accordingly, we introduced a long transient phase, which ensured that this condition holds. With respect to $\delta_{i}$, it affects the stability of $x_{i}\left(t\right)$ in time, determining how much it can vary from round to round: increasing $\delta_{i}$ makes $x_{i}\left(t\right)$ more stable and vice versa. In order to nullify the influence of $\delta_{i}$, we did not simply analyze the final value of $x_{i}\left(t\right)$, but its average value after the transient phase.

The ISB is definitely the most interesting part, since in this experiment we exploited it to study its effect on the citizens’ opinion. In such a context, $use_{ij}\left(t\right)$ is represented by the frequency of use of the sources. For instance, if *i* has a frequency of use of 50% for source *j*, this means that there is the 50% probability that *i* will access this source at time *t*. For what it concerns the weights given to the reported information, $a_{ij}$, this can be generate by the trust value *i* has on *j* for reporting information about COVID-19. For the sake of simplicity, we used already normalized weights, as in Equation (4) (of course, given Equation (4), it is also necessary that $\delta_{i}\neq 1$; otherwise, $x_{i}\left(t\right)$ will not be affected by information sources). Specifically, we keep $\delta_{i}$ fixed, and we assign the weights of the other sources proportionally to their trust values. The choice to use trust values as weights for information source has a solid foundation in the literature [31,32,33] for other models and specifically for Hegselmann–Krause [34]. All the data we need to generate $use_{ij}\left(t\right)$ and $a_{ij}$, the frequency of use and the average trust, can be found respectively in Table 2 and Table 3.

We come therefore to the last parameter of the experimental setting, the actual trustworthiness of the information sources. Unfortunately, there is no way to find such data. To the best of our knowledge, we could not find any study about it, which is reasonable, since it is not that easy to produce a precise quantification of how trustworthy these information sources were during the pandemic. Nevertheless, two possible approaches allowed us to overcome the problem. The first one consists of equalizing trust (the assessment of the trustor) and trustworthiness (the intrinsic property of the trustee, determining its actual performance). Given that it is rarely possible to directly access the effective value of trustworthiness, we may reasonably suppose that the assessment of the survey respondents approximates sufficiently well the real trustworthiness of the information sources. To give a clearer idea, this is exactly what currently happens in systems such as Google, Ebay or TripAdvisor: the evaluation of a seller/restaurant/hotel is obtained by aggregating the evaluations produced by a multitude of individuals; this is not the real trustworthiness, but it is an appropriate approximation. In this specific case, we easily extrapolated such data from the questionnaire. We believe that this first approach (average trust value—ATV), with a certain margin of unavoidable error, represents the most precise solution.

As an alternative, it is possible to arbitrarily rank the sources on the basis of their perceived trustworthiness (trustworthiness-based sorting—TBS), assigning the specific values accordingly. In the ATV case, these values approximately vary form a minimum of 20% to a maximum of 90%. We consider the same ranking and the same range in the TBS case, distributing the 6 sources in intervals of equal dimensions. In this study, we took into account both the possibilities.

As already stated, the trustworthiness of a source determines $inf_{j}\left(t\right)$; i.e., it represents the actual probability that the source *j* will report information supporting the belief *b*, at time *t*. For instance, in the ATV case, scientists will report supporting information ($inf_{j}\left(t\right)=1$) 88.85% of the time, and opposing information ($inf_{j}\left(t\right)=0$) the rest of the time.

Considering the workflow of the simulation, at the beginning of each run, we found the various information sources, each described by a specific value of trustworthiness, and a number of citizens. Each citizen *i* belongs to a specific category, which in turn determines its ISB. At each round of this phase:

1. The sources report fresh information supporting *b* or opposing to it;
2. The citizen *i* accesses a subset of the available information sources, according to its ISB, and updates its opinion $x_{i}\left(t\right)$ according to the formula 3.

The simulation consists of two different parts: the transient phase, whose sole function is to ensure that the citizen’s initial profile will not affect $x_{i}\left(t\right)$ in the next phase; the analysis phase, in which we actually analyze the value of $x_{i}\left(t\right)$. In particular, we are interested in the average value of $x_{i}\left(t\right)$ during this last phase, which is used, within this framework, as a performance indicator. Ideally (for the institutions), this dimension should tend towards the target value 1 (the citizen believes *b* to be true), while low values are undesirable (the citizen does not believe *b*).

The resulting model has been implemented in the simulation environment NetLogo (version 5.2) [35], developed by Northwestern University Center for Connected Learning and Computer-Based Modeling (Evanston, IL, USA). We provide further details about the implementation in the specific Section 4.4.

## 4. Results

### 4.1. Descriptive Statistics

In this section, we investigated both frequency of use and perceived trustworthiness of various types of information sources in relation to the COVID-19 pandemic, to get a better sense of what channels were most influential in affecting participants’ opinions on this topic; in addition, we collected data on the trustworthiness directly assigned to public institutions as information sources.

**Figure 2:**
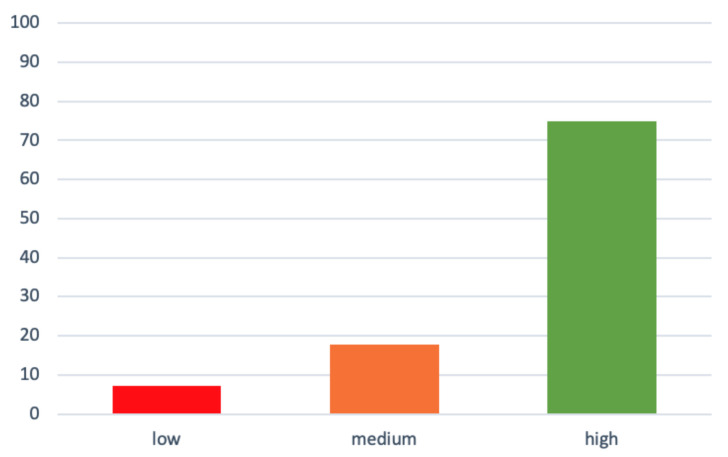
Trust in public institutions for the management of COVID-19.

We start our considerations from trust, since this is the key concept around which the response of the Italian population revolves, thereby affecting their decisions to accept guidelines and prescriptive measures. When we asked the subjects to rank their overall trust in the institutions for the management of the COVID-19 emergency, 75% of respondents manifested either extreme (23.8%) or high (51.2%) levels of trust, 17.7% were non-committal, and only 7.3% expressed distrust (see Figure 2). These numbers are in sharp contrast, to say the least, with the average institutional trust reported for Italian citizens prior to the COVID-19 crisis.

Of course, the various sources of information that people used had a strong influence on what they believed and thought, and thus also on their perception of the authority’s trustworthiness. It is therefore essential that citizens make proper use of the various sources of information they can access, as this may in turn determine their decisions to adopt the rules imposed by the authorities.

Then, we analyzed the information seeking behavior (ISB) of the respondents, with particular reference to the following sources of information:

- Traditional media;
- Official websites;
- Social media;
- Family physicians;
- Scientists;
- Friends, relatives, acquaintances (f.r.a.).

The collected data (Table 4) give us a clear picture about people’s perceptions of sources’ trustworthiness. About 92.6% of the respondents reported to trust scientists. Immediately after, 89.6% trusted official websites. Then followed family physicians (63%), traditional media (41.7%), and at a quite significant distance, f.r.a. (7.3%) and social media (4.3%). Such a ranking points out a somewhat rational picture, with respect to the actual performance of the sources and also to those who produce or report information: starting from scientists, then official channels, finishing with social media.

What is indeed different and unusual, also with respect to the state of the art [28,36,37], is the use the citizens made of information channels. We would have expected to find, among the most used sources, f.r.a., traditional media or even social media. These may not be the most reliable sources, though they are easily accessible and commonly used. Instead, we observed a strong and unexpected change regarding the use of information sources, probably related to the pressing need for reliable information.

It is definitely striking to find that the most used sources, immediately after the traditional media (78.7%), were scientists (70.6%) and official websites (77.8%). Other secondary variables may have influenced the actual frequency of use, such as the ease of use, or the fact that the same source was used for different purposes. This may hold for traditional media, but it is certainly not true for scientists and official websites. Another factor that may have influenced the use of the sources is their very trustworthiness. Indeed, we identified a high correlation (Table 5) between believing that a specific source is reliable and actually using it. In other words, to a reliable source corresponds a greater use, while to an unreliable source corresponds a lower use. Such an effect appears to be strong for all the information sources, and it is particularly evident for the least reliable sources: f.r.a. (R = 0.643, *p* < 0.0001) and social media (R = 0.642, *p* < 0.0001). Thus, it is possible that the critical situation, together with the necessity to introduce efficient control mechanisms, may have determined the strong use of reliable sources.

To summarize, the results of this section highlight four main findings: (i) official websites, e.g., the website of the Civil Protection, and scientists are both frequently used and considered reliable as information sources; (ii) in contrast, traditional media, albeit often consulted, are regarded as reliable only by less than half of our sample; (iii) family physicians are in general considered trustworthy, yet they are rarely used as information sources; (iv), finally, both social relationships and unofficial online sources, e.g., social media, are neither frequently used, nor widely believed.

### 4.2. Short-Term Shifts Over Time

We report a slight, though not astonishing, influence of time on the behavior of individuals. In relation to the introduction of more restrictive measures by the Italian Government on 11 March, we divided our sample based on time of data submission: before and after the public press release when the Prime Minister Giuseppe Conte announced the new restrictions to be implemented nationwide, to contain the COVID-19 outbreak.

Indeed, this seems to have slightly affected both trust and information need (see Table 6). Except for family physicians, whose use decreased and for whom trust remained stable over time, the use (from 56.7% to 58%) and trust (from 60.7% to 61.7%) increased for all the other information sources. These results provide further evidence of the already identified "trust gap": the greater the trust required to implement the containment measures, the greater the trust that individuals granted. As a further consequence, the need to acquire more information also increased. In other words, when a further restrictive measure resulted, in this case, in a further increase of information was requested.

### 4.3. Effects of Age and Gender

Comparing male and female respondents, significant differences emerged concerning their ISB. We identified an average 4% higher information request for women, with respect to men. Such a difference increases when we consider the use of online sources. There is an increment of +6.6%, confirmed by correlation data, both for official websites (R = 0.124, *p* < 0.0001) and social media (R = 0.1, *p* < 0.0001). This effect is well-supported by previous evidence in the literature, since other studies detected a higher tendency of women to refer to online sources for health information [37,38]. This effect disappears when we consider scientists as information sources (there is a slight difference of −0.7%). On the contrary, we did not find significant gender effects about trust.

Investigating the effects of age, we also considered a 10-year range re-coding. The idea that led to the categorization process was to investigate the behavior of individuals subjected to the same level of risk (death rate). In this regard, we refer to the most common death rate classifications (age, sex, existing conditions of COVID-19 cases and deaths, https://www.worldometers.info/coronavirus/coronavirus-age-sex-demographics/; accessed at 15/03/2020) [24], considering age in 10-year ranges. As far as it concerns age, young people tend to consult information sources with a lower frequency, compared to older people, going from an average frequency of use of 53.86% for 18–29 to a 61.41% for people over 70. The different levels of risk that COVID-19 entails between young and old people [23,24], in particular in the severity it can take, could at least in part explain the differences introduced by age regarding the willingness to keep informed about this phenomenon. Young people were also the most suspicious with respect to sources of information: 30–39 year-old people showed the lowest level of trust (57.71%, slightly higher than 18–29), which increased with age until 64.03% for people over 70.

Combining the effects of age and gender (Table 7), we found that 18–29 year-old men had the lowest average frequency of use (52.9%) while 30–39 year-old men hde the lowest level of trust (58.9%). In contrast, over 70 year-old women were the most prone to use information sources (63.3%) and to trust them (68.2%).

Table 2 reports the frequency of use for the different information sources. Focusing on the least trusted ones, we observed that people over 70, more exactly women, are the ones that make most use of f.r.a. and social media. The people over 70’s average use of social media was 37.4%, and it even increased up to 49.2% for women. Advanced age does not seem to have represented a barrier to accessing social media. This result is even more striking when crossed with the analysis of trust (Table 3). The average trust in social media is about 20%, but it rises to 29% for men over 70, and even to 39% for women over 70. Summarizing, on the one hand, women over 70 make extensive use of information sources; on the other hand they show a higher level of trust in social media, which certainly cannot be considered the most reliable source. A deeper analysis of these two contradictory characteristics may offer some interesting insights.

### 4.4. Simulations

The analysis presented so far, based on the data collected through the questionnaire, already provided us with interesting ideas and results suggesting a partial explanations to many of the behaviors occurred in Italy, during the first phase of the pandemic. Starting from those findings, in the following experiments we considered a social simulation by implementing a model that allowed us to get further relevant estimates. Specifically, we were interested in:

- Determining how much the ISBs of the different categories of citizens, classified by age and gender, affected their opinions, and in turn, their choices during the pandemic.
- Trying to compare the ISBs identified in this study with those prior to COVID-19 arrival. Such a comparison may help determine whether and to whay extent the citizens’ rational and responsible choice to rely on trusted sources positively affected their acceptance of restrictions and rules needed to face the pandemic.

#### 4.4.1. First Experiment: The Influence of ISBs on the Citizens’ Opinions

The analysis in Section 4.3 suggests that women and older people show a greater interest in information (regarding the pandemic), while young men seem to be the most disinterested category. That being said, it is still necessary a more in-depth analysis to understand how these characteristics affected the opinion of the individuals, making them more or less resilient to misinformation. Especially for women over 70, we thought it interesting to verify how their frequent usage and high trust in social media have affected their opinions, and whether their higher use of the other sources can compensate for these contrasting characteristics. More generally, it is particularly useful to quantify the impacts of information sources on the various categories of citizens. Consider, for instance, that being able to study and to quantify the evolution of their opinion would allow the institutions to identify which part of the population needs targeted interventions, offering the possibility to optimize the available resources.

While aiming to quantify those dimensions, within this section we introduce an agent-based simulation, which allows us to evaluate how the citizens’ opinion changes depending on their ISBs. In particular, we considered an opinion dynamics model based on that proposed by Hegselmann–Krause [30]. Their model is used to investigate opinion dynamics within a group of individuals who interact with each other by exchanging opinions. Within this experiment, we focus on the opinions of individuals, in a context in which they receive information from their sources in a unidirectional way (the source reports information to the individual, but the opposite does not happen). Conversely, in the next experiment we consider the opinion of the whole population.

We report below the experimental setting:

1. Transient phase: 2000 rounds. Actually, we verified that a significantly lower number of rounds would be sufficient. Yet, we decided to use a high value.
2. Analysis phase: 100 rounds.
3. $x_{i}\left(0\right)$: 0.5. As we stated, this parameter did not affect the final values.
4. $\delta_{i}$: 100%. As we stated, this parameter did not affect the final values.
5. Number of citizens: 1200, i.e., 100 for each category.
6. Frequency of use in Table 2.
7. Trust in information sources in Table 3.
8. Trustworthiness of the information sources for the ATV and TBS cases in Table 8.

The data summarized in Table 9 show the findings of the experiment, both in the ATV case and the TBS case. Overall, we observe a positive tendency, as the average opinion of all the categories of citizens is over the threshold 0.5. This result was driven by the intensive use of reliable information sources, first, above all, scientists. Nevertheless, it is worth noting that in none of the cases analyzed did we encounter a striking value, particularly close to the target value 1.

Coming then to detail, it is plainly clear that women over 70 had the worst performance. Their average opinion is 0.661 in the ATV case and 0.628 in the TBS case.

In contrast, 30–39 year-old men showed the best performance: their average opinion was 0.731 in the ATV case (+7% with respect to the worst case) and 0.69 in the TBS case (+6.2% with respect to the worst case).

#### 4.4.2. Second Experiment: A Comparison between the Citizens’ ISBs before and after the Arrival of COVID-19

All the data and the analyses previously reported in this work clearly suggest a substantial virtuous behavior of the Italian population. In particular, in the attempt to get reliable information, there has been an extraordinarily high reliance on science. We can then assert that this virtuous behavior actually played an important role in the fight against the pandemic: being properly informed encouraged the acceptance of the rules and guidelines, despite the high personal burden.

It would be interesting to quantify to which extent this virtuous behavior has helped to reduce the impact of COVID-19. In this sense, our effort within this second experiment was to compare the estimation of the public opinion in this case with what would have happened if the population had behaved, with respect to information, as it usually did in health-related contexts before COVID-19. We made use of the same framework of the previous experiment. If, however, we previously evaluated the individual categories, here we were interested in studying the opinion of the whole population. In other words, we implemented the logic of the previous experiment, considering a whole community, made up of citizens belonging to the same categories. In order to obtain a more likely outcome, we took into account the real distribution of the Italian population, according to age and gender. Such data, current as of January 2019, have been retrieved from the Istat website (http://dati.istat.it/Index.aspx?QueryId=42869 accessed at 07/08/2020). Table 10 summarizes them.

We considered a population of 1000 citizens, which allowed for a more precise distribution of the citizens among the categories. Then we analyzed the average opinion of the whole population. We compared the results obtained with the ISBs extrapolated from our survey (outbreak setting—OS), concerning the COVID-19 outbreak, with the one (prior setting, PS) retrieved by the fairly recent study of Zucco and colleagues [37], conducted in May 2017 in Italy (N = 913). The study aimed to identify the ISB concerning antibiotics and health in general. Even if it does not provide all the data we needed concerning health in general, it does for antibiotics. Moreover, at least for what concerns the available data, the frequencies of use appear quite similar in these two contexts. Thus, we considered data about information sources’ usage for antibiotics (Table 11). We considered the same population of 1000 citizens, verifying how the average opinion changed when introducing the ISBs of Italian population before COVID-19.

Summarizing the experimental setting:

1. Transient phase: 2000 rounds. Actually, we verified that a significantly lower number of rounds would be sufficient. Yet, we decided to use a high value.
2. Analysis phase: 100 rounds.
3. $x_{i}\left(0\right)$: 0.5. As we stated, this parameter did not affect the final values.
4. $\delta_{i}$: 100%. As we stated, this parameter did not affect the final values.
5. Number of citizens: 1000, distributed by age and gender according to Table 10.
6. Frequency of use: Table 2 for OS agents and Table 11 for PS agents.
7. Trust in information sources in Table 3.
8. Trustworthiness of the information sources in Table 8.

Results in Table 12 highlight a substantial difference of opinion. Specifically, OS agents had +8.2% and +9.1% higher outcomes, respectively, in the ATV and TBS cases. These findings suggest that the responsible behavior of the population, with respect to information, has indeed helped the acceptance of the rules imposed by the institutions and it may have contributed to reducing the impact of COVID-19.

## 5. Discussion

In this study, we focused on the ISB of the Italian population during the early stages of the pandemic. In a situation of high risk and uncertainty, accurate information becomes priceless. This is what happened in Italy, in the early stages of the COVID-19 pandemic. On the one hand, there was the growing need to be informed about what was happening in order to understand the general characteristics, the risks, and the evolution of the phenomenon. On the other hand, information represented a way to check and control the actions of the institutions, to check that everyone’s personal sacrifice was not in vain, that the granted trust was not misplaced. Even the World Health Organization, in a 2011 report [7], identified communication as one of the biggest challenges to tackle a pandemic. We found confirmation of this in our analysis, detecting a strong correlation (R = 0.545, *p* < 0.0001) between trusting institutions for managing the COVID-19 emergency and as a source of information for the same topic.

From the need to rely on the institutions, in a historical context of substantial distrust, followed the necessity to introduce control mechanisms to fill this trust gap, to supervise somehow the actions of the institutions. What we detected here is a well known phenomenon in the literature [5,39]. When the trustor (citizens) does not trust enough the trustee (Italian institutions), it can decide to introduce control mechanisms to check the trustee’s actions. Thus, the trustor (to realize the practical act of trusting) needs a lower level of trust (as mental attitude). In our specific case, to trust the institution was the only possible choice; thus, the higher information request [2] was introduced as a control mechanism.

This affected not only the quantity, but also the quality of the information requested. Indeed, we detected significant changes with respect to people’s behavior towards information. Such changes may have positively affected the effectiveness of the measures introduced to tackle the pandemic, facilitating their acceptance by the whole population. The most remarkable result is probably the very high usage of scientists as information sources. About 92.6% of the respondents reported to trust scientists. This is definitely far from what has been found in other studies [37], especially if we consider the marginal role science has played in Italy over the last few decades. It is worth noting that, before COVID-19, Italy had a fairly worrying scientific picture, both from the political and the societal perspective (decreasing funding for the sector, and citizens’ distrust towards scientific rationality: think of phenomena such as flat-Earthers or anti-vaxers). After the COVID-19 outbreak, science has suddenly recovered an important role in the citizens’ consideration. This is quite reasonable because the only concrete answer (both as analysis of the phenomenon and as ability to mitigate and oppose to it by new tools and approaches) was the one provided by science and scientific research.

As far as it concerns age and gender, we found that they significantly affect the ISB. We identified a higher average number of information requests for women, with respect to men. This difference becomes particularly evident for online sources. Such a higher tendency of women to refer to online sources for health information has already been identified in the literature [37,38]. Given the fundamental role of information in this context, knowing how individuals inform themselves (and possibly the specific categories to which they belong) and how they use information can be fundamental to understanding how to promote wider and more balanced information.

It is crucial to specifically analyze how citizens relate to social media. This is because it is perhaps the most dangerous source of information. Larson [40] even states that, "The deluge of conflicting information, misinformation and manipulated information on social media should be recognized as a global public-health threat." Allington and colleagues [41] confirm that it is also dangerous for this specific pandemic.

In our study, for example, it is interesting to note the case of women over 70 compared to other categories: they make a particularly relevant use of social media. This is alarming, since the category subjected to the higher risk is also the one that relies most on those that are widely identified as the least reliable media. Experiments confirm that this characteristic has a strong negative impact on women over 70, such that their average opinion is the lowest ever (compared to the institutional point of view). Remarkably, it is a serious problem that one of the groups subjected to the highest risk [42,43] relies that much on what looks like the worst information source (at least from the institution’s point of view). Therefore, such a negative performance is not related to lack of information, but rather to a lower ability to relate to information [44]: they probably do not know they should distrust social media.

We found that young people, and particularly young men, tend to consult information sources with a lower frequency, compared to older people. Such a characteristic can be traced back to different factors. One of them is information avoidance. Over the years, researchers have spotlighted the human tendency to avoid, ignore, and/or deny information, particularly in the context of health care, if paying attention to it will cause mental discomfort or dissonance [45]. As Maslow [46] stated: “We can seek knowledge in order to reduce anxiety and we can also avoid knowing in order to reduce anxiety.” Sometimes we would rather not know that we are at high risk for a disease or disaster. Moreover, it could be that keeping oneself informed would put one in conflict with one’s other goals: if a person needs to go to work, they might prefer not to know that going to work puts them at risk by choosing not to look for new information. Another possibility is that young people felt less involved than the elderly, given the great difference in mortality. Of course, many other reasons could explain this effect: less time available; being engaged in other activities; the fact that young people, unlike the elderly, had also the problem of losing their jobs. In any case, this difference seems to be abundantly compensated by a conscious use of information sources. In light of their better result, it seems that a "less but better" strategy is in this case much more rewarding. To conclude, getting more information is not always synonymous with being better informed. Although keeping informed was crucial in the first phase of the pandemic, it is also necessary a proper approach to information.

Last but not least, in this work we compared the ISB detected during the pandemic with that detected in other studies before the arrival of COVID-19, also in Italy. Indeed, it appears that the exceptionally responsible use of information sources has had a positive effect on the opinion of the population (+8.2% and +9.1% higher, respectively in the ATV and TBS cases). Such a difference is particularly critical, as it concerns the average opinion of the population. Consider, in fact, that the effective success of the rules introduced to deal with COVID-19 depended on the adoption of these rules by a significant percentage of the Italian population. Therefore, a lower average opinion was not just a symptom of a lower acceptance level, but it could have involved a chain reaction, discouraging even those who considered such measures useful. Such a situation was clear both to citizens and institutions: the perceived utility of these measures positively correlated with believing that a sufficient number of individuals will follow these restrictions (R = 0.233, *p* < 0.0001) and negatively correlated with believing that an insufficient number of people will do that (R =−0.21, *p* < 0.0001). In view of these findings, we may conclude that the responsible behavior we detected in this study, with respect to information, has indeed helped the acceptance of the strict and restrictive rules imposed by the institutions and it may have contributed to reducing the impact of COVID-19.

## 6. Conclusions

The initial survey [4], on which this work is based, has reported various insights and evidence. In particular, a significant increase in citizens’ trust in public authorities was witnessed: "The analysis focused on how citizens attribute trust to public authorities, in relation to the management of the health crisis: with regard to the measures and guidelines adopted, the purposes pursued, the motivations that determine them, their capacity for involvement and their effectiveness for the containment of the virus itself." In this work, we investigated the relationship between COVID-19 and the citizens’ need for information. This need for information explains the disproportionate increase in trust that we have witnessed (and that the survey accurately reported). In fact, a verification and control mechanism came into play, acting as a compendium (and as a rational and non-fideistic moderator) to the disproportionate trust we have witnessed (and which proved to be necessary to face such a delicate phase). Verifying and controlling the development of the epidemic and the countermeasures put in place with their potential effects and findings represented a particular input that is clearly captured by this study which defines its characteristics and methods.

This study shows how the role of information was fundamental in dealing with COVID-19. Thanks to the possibility of obtaining information and comparing the different sources of information, citizens have been able to appreciate the fundamental role played by science in modern societies. Obviously, even the information coming from scientists has had a dynamic (with some contradictory positions) strictly linked to the awareness that science has acquired over time on the phenomenon in question. Our hope is to witness a maturation of public opinion towards the sources of information that persist over time. Moreover, although the critical phase of the pandemic seems to be over, it is essential to continue monitoring its evolution, taking care of the complex relationship between the institutions and citizens. We sincerely hope that the considerations included in this work, in addition to clarifying what has happened in the past months and what has worked or not, can also help public institutions and the healthcare system in the future.

It is worth noting that this study is not without limitations. Most notably, given that the sample was predominantly composed of individuals with a high level of education, it is possible that some associations could not be detected because of the lack of variability in the study sample.

To conclude, we reserve for the future attempts to deepen some aspects that we left out in this work. We have not analyzed the effect that the rapidly changing, at times also contradictory, hypotheses proposed by the scientific community had on the population. The reason for this lack is that this effect did not arise in the considered time window, but immediately after. Furthermore, we have not taken into consideration the health status of the subjects (presence of pre-existing diseases), which could have provided interesting insights.

## Figures and Tables

**Table 1:** Sample characteristics.

	Regions Most Affected % (30%)	Regions Less Affected % (70%)	Total %
*Gender*
Men	45	42	43
Women	55	58	57
Total	100	100	100
*Age (Mean = 46)*
18–29	19	11	13
30–39	23	18	19
40–49	23	24	24
50–59	21	28	26
60–69	11	15	14
>70	3	4	4
Total	100	100	100
*Educational Level*
Middle school	3	2	2
High school	24	27	26
University degree	41	36	38
Post-graduate specialization	32	35	34
Total	100	100	100
*Geographical provenance*
Northern Italy	96	7	33
Central Italy	4	53	39
Southern Italy/islands	0	40	28
Total	100	100	100

**Table 2:** Frequency of use for each information sources, based on age and gender.

Category	Traditional Media	Official Websites	Social Media	Family Physicians	Scientists	F.r.a.
women 18–29	77.82%	78.61%	42.24%	31.58%	59.56%	42.32%
women 30–39	75.67%	83.73%	41.94%	32.54%	67.08%	38.31%
women 40–49	82.33%	83.77%	39.92%	38.86%	74.53%	35.17%
women 50–59	84.75%	82.19%	37.81%	39.82%	76.93%	35.97%
women 60–69	87.38%	80.05%	38.88%	46.21%	81.31%	37.15%
women over 70	91.25%	61.25%	49.17%	48.33%	79.17%	50.83%
men 18–29	68.85%	75.81%	35.79%	25.20%	66.94%	39.01%
men 30–39	73.44%	78.28%	31.80%	26.39%	69.92%	34.75%
men 40–49	76.89%	77.24%	34.67%	30.84%	72.82%	31.96 %
men 50–59	80.14 %	74.79%	34.53%	36.76%	75.95%	30.56%
men 60–69	86.84%	70.94%	29.77%	47.88%	78.62%	33.04%
men over 70	88.41%	65.85%	35.98%	54.88%	75.00%	39.94%

**Table 3:** Trust in information sources, based on age and gender.

Category	Traditional Media	Official Websites	Social Media	Family Physicians	Scientists	F.r.a.
women 18–29	55.09%	89.73%	19.83%	64.66%	88.56%	29.78%
women 30–39	54.53%	87.88%	19.27%	64.79%	86.78%	30.34%
women 40–49	55.72%	87.29%	20.34%	69.32%	90.04%	30.04%
women 50–59	57.63 %	86.64%	20.72%	69.54%	89.82%	32.63%
women 60–69	59.38 %	86.04%	21.45%	71.77%	91.32%	34.70%
women over 70	65.42 %	84.58%	38.75%	75.42%	90.42%	48.75%
men 18–29	51.01 %	87.60%	20.87%	66.23%	88.51%	30.24%
men 30–39	50.33 %	85.57%	16.39%	66.64%	87.38%	28.85%
men 40–49	55.25 %	84.73%	18.51%	69.93%	87.38%	28.54%
men 50–59	56.46 %	82.15 %	18.33%	69.70%	88.56%	29.56%
men 60–69	59.19 %	81.54%	19.43%	70.94%	89.13%	31.27%
men over 70	60.06 %	77.44 %	28.96%	73.17%	91.16%	39.33%

**Table 4:** Use and reliability of information sources.

SOURCE		USE			TRUST	
	Frequent	Average	Infrequent	Trustworthy	Neutral	Untrustworthy
Traditional media	78.7	11.9	9.2	41.7	38.7	19.6
Official websites	77.8	12.2	10	89.6	8.1	2.3
Social media	25.6	18	56.5	4.3	17.7	78
Family physicians	24.6	20.1	55.2	63	26.3	10.7
Scientists	70.6	15.6	13.8	92.6	6.2	1.2
F.r.a	16.6	29.2	54.2	7.3	33.2	59.5

**Table 5:** Correlation between trust and actual use of a source of information.

Source	R	*p*
Scientists	0.396	<0.0001
Official websites	0.253	<0.0001
Family physicians	0.376	<0.0001
Traditional media	0.469	<0.0001
F.r.a.	0.643	<0.0001
Social media	0.642	<0.0001

**Table 6:** Use and reliability of information sources before and after the announcement of more restrictive measures.

SOURCE	USE		TRUST	
	Before	After	Before	After
Traditional media	79.9	81.8	55.6	57.2
Official websites	78.5	80.2	85.5	87
Social media	36.3	42	19.5	22.1
Family physicians	37.2	33.9	68.6	68.6
Scientists	72.8	73.2	88.8	89
F.r.a	35.6	36.9	31	31.2

**Table 7:** Average values of frequency of use and trust in information sources, based on age and gender.

Category	Average Use	Average Trust
women 18–29	55.36 %	60.85%
women 30–39	56.54%	60.02%
women 40–49	59.10%	61.30%
women 50–59	59.58 %	61.99%
women 60–69	61.83%	63.27%
women over 70	63.33 %	68.15%
men 18–29	51.93%	59.85%
men 30–39	52.43%	58.86%
men 40–49	54.07%	59.94%
men 50–59	55.46%	59.78%
men 60–69	57.85%	60.68%
men over 70	60.01%	63.11%

**Table 8:** Trustworthiness of the sources of information.

Source of Information	ATV	TBS
scientists	88.85%	90%
official websites	85.82%	76%
family physicians	68.63%	62%
Traditional media	55.91%	48%
f.r.a.	31.04%	34%
social media	20.02%	20%

**Table 9:** Average values of the citizens’ opinions during the analysis phase. The citizens are grouped by age and gender. We report average values over 100 simulations.

Category	Average Opinion—ATV	Average Opinion—TBS
Women 18–29	0.704	0.661
Women 30–39	0.719	0.677
Women 40–49	0.723	0.682
Women 50–59	0.723	0.683
Women 60–69	0.715	0.677
Women over 70	0.661	0.628
men 18–29	0.718	0.679
men 30–39	0.731	0.69
men 40–49	0.726	0.686
men 50–59	0.723	0.684
men 60–69	0.716	0.677
men over 70	0.69	0.654

**Table 10:** Distribution of the Italian population by age and gender, current as of January 2019.

Category	Men	Women
18–29	7.55%	7.01%
30–39	7.02%	6.94%
40–49	9.06%	9.17%
50–59	9.03%	9.42%
60–69	6.93%	7.55%
over 70	8.55%	11.76%

**Table 11:** Frequency of the use of information sources extrapolated from the study of Zucco and colleagues.

	Traditional Media	Official Websites	Social Media	Family Physicians	Scientists	F.r.a.
frequency of use	13.6%	27.6%	45.1%	71.6%	8.9%	1.7%

**Table 12:** Average opinion of the OS and PS populations, in the ATV and TBS cases.

	Average Opinion—ATV	Average Opinion—TBS
OS	0.71	0.664
PS	0.628	0.573

## Abbreviations

The following abbreviations are used in this manuscript:

ATV	average trust value
f.r.a.	friends, relatives, acquaintances
ISB	Information Seeking Behavior
OS	outbreak setting
PS	prior setting
TBS	trustworthiness-based sorting
